# Complete Genome Sequence Analysis of *Bacillus subtilis* Bbv57, a Promising Biocontrol Agent against Phytopathogens

**DOI:** 10.3390/ijms23179732

**Published:** 2022-08-27

**Authors:** Raguchander Thiruvengadam, Karthikeyan Gandhi, Sendhilvel Vaithiyanathan, Harish Sankarasubramanian, Karthiba Loganathan, Rajendran Lingan, Veera Ranjani Rajagopalan, Raveendran Muthurajan, Jonathan Ebenezer Iyadurai, Prabakar Kuppusami

**Affiliations:** 1 Department of Plant Pathology, Tamil Nadu Agricultural University, Coimbatore 641 003, Tamil Nadu, India; 2 Department of Plant Biotechnology, Tamil Nadu Agricultural University, Coimbatore 641 003, Tamil Nadu, India; 3 Department of Nematology, Tamil Nadu Agricultural University, Coimbatore 641 003, Tamil Nadu, India

**Keywords:** *Bacillus subtilis*, Bbv57, whole genome sequence, biocontrol agent, PGPR, secondary metabolites

## Abstract

Plant growth-promoting rhizobacteria (PGPR) are a group of root-associated beneficial bacteria emerging as one of the powerful agents in sustainable plant disease management. Among the PGPR, *Bacillus* sp. has become a popular biocontrol agent for controlling pests and the diseases of several crops of agricultural and horticultural importance. Understanding the molecular basis of the plant growth-promoting and biocontrol abilities of *Bacillus* spp. will allow us to develop multifunctional microbial consortia for sustainable agriculture. In our study, we attempted to unravel the genome complexity of the potential biocontrol agent *Bacillus subtilis* Bbv57 (isolated from the betelvine’s rhizosphere), available at TNAU, Coimbatore. A WGS analysis generated 26 million reads, and a de novo assembly resulted in the generation of 4,302,465 bp genome of *Bacillus subtilis* Bbv57 containing 4363 coding sequences (CDS), of which 4281 were functionally annotated. An analysis of 16S rRNA revealed its 100% identity to *Bacillus subtilis* IAM 12118. A detailed data analysis identified the presence of >100 CAZymes and nine gene clusters involved in the production of secondary metabolites that exhibited antimicrobial properties. Further, Bbv57 was found to harbor 282 unique genes in comparison with 19 other *Bacillus* strains, requiring further exploration.

## 1. Introduction

Plant diseases due to fungi, bacteria, viruses, *Candidatus phytoplasma*, fastidious vascular bacteria, and viroids cause an estimated yield loss of 14% in diverse crops of agricultural importance, leading to an economic loss of 220 billion U.S. dollars [1] The transboundary movement of pathogens introduces new diseases in several geographical locations, which poses a threat to global food security [2]. To sustain crop productivity against diseases, chemicals are used, which is inadvertently responsible for environmental pollution and health hazards [3]. In this context, the cultivation of resistant varieties and use of biocontrol agents will minimize the use of synthetic chemicals. This approach will protect the environment in addition to sustaining ecological balance. The rhizosphere harbors beneficial microorganisms that have potential to be used as biopesticides in plant disease management and to induce systemic resistance in the host [4]. These rhizospheric bacteria such as *Bacillus*, which belongs to the family Bacillaceae, were found to contain bioactive molecules with growth-promoting activity and antagonistic effects against phytopathogens [5]. Its faster growth rate and resistance to adverse environmental conditions through the production of endospores have made *Bacillus* a popular biocontrol agent [6]. *Bacillus* was also reported to produce volatile compounds exhibiting growth promotion and triggering defense mechanisms in plants [7,8].

In one of our earlier studies, a potential bioinoculant, *Bacillus subtilis* Bbv57, exhibiting fungicidal/bactericidal/nematicidal properties was isolated from a betelvine’s rhizosphere (accession No. MW282917; [9]. *B. subtilis* Bbv57 is a Gram-positive rod-shaped bacterium arranged in pairs or chains with rounded or square ends, usually has a single endospore, and is able to grow between 4 °C to 45 °C. It utilizes citrate, hydrolyze starch, and gelatin while reducing nitrate. The presence of genes encoding antimicrobial peptides, viz., iturin (*ItuD*), surfactin (*srfA*; *sfp*), bacilysin (*bacAB*; *bacD*), bacillomycin D (*bamD*), fengycin (*fenB*), ericin (*eriB*), mycosubtilin (*mycC*), and subtilin (*spaB*), was analyzed in *B. subtilis* Bbv57 through PCR. Additionally, the presence of two quorum-sensing genes, *aiiA* and *comQ*, was also reported [9,10]. The isolate synthesizes hydrogen cyanide (HCN), IAA, GA3, SA, siderophore, protease, exopolysaccharides, and biofilm, and it possesses intrinsic antibiotic resistance to ampicillin, erythromycin, and clindamycin and intermediate resistance to cephalothin and oxacillin. A bioassay using the crude extract of Bbv57 revealed its antagonistic effect against *F. oxysporum* and *Meloidogyne incognita* in gerbera and increased flower yield by 23.36% [9,11]. The crude lipopeptide antibiotics of Bbv57 exerted lethal effects on the eggs and juveniles of the root-knot nematode for up to 72 h of exposure, compared with that of a control [12,13]. The conserved ITS region 16SrRNA of Bbv57 was amplified with an amplicon size of 1460 bp, sequenced, and deposited in the NCBI database (Accession No. MW282917). However, whole genome sequencing (WGS) of the isolate Bbv57 unravels the molecular basis of its plant growth-promoting and antimicrobial properties. The efficiency of WGS in differentiating some closely related *Bacillus* sp. was reported earlier [14]. Further, WGS may also enable the identification of carbohydrate-active enzymes (CAZymes) and secondary metabolites that play a major role in biocontrol properties [3,11].

Sophisticated bioinformatics tools like SMURF and antiSMASH have powered the identification of biosynthetic gene clusters (BGCs) and secondary metabolite gene clusters (SMGC) [15,16,17,18]. Based on the above facts, our study aimed to unravel the genome complexity of *Bacillus subtilis* Bbv57 to identify the genetic factors underlying its plant growth-promoting and biocontrol properties. WGS, combined with a detailed bioinformatics analysis, identified novel gene clusters in Bbv57 that encoded for CAZymes and secondary metabolites. This study provides insight into the genome of *B*. *subtilis* Bbv57 and thus exploits its genetic potential in future research. 

## 2. Results

### 2.1. Genomic Features of Bacillus subtilis Bbv57

The whole genome sequencing of *B. subtilis* Bbv57 yielded 4,302,465 bp with an average G + C content of 44.5%, five copies of the rRNAs operon (16S, 23S and 5S RNA), and 76 tRNA genes. The Bbv57 genome was predicted to contain 4363 coding sequences (CDS), of which 4281 were functionally annotated (Table 1). All the protein-coding genes were assigned to COGs (cluster of orthologous groups). The functional classes defined by COGs indicated that *B. subtilis* Bbv57 harbors a high proportion of proteins involved in amino acids transport and metabolism (COG E) and transcription (COG K), followed by carbohydrate transport and metabolism (COG G). Sixty-six different protein-encoding genes were found to be involved in defense mechanisms (COG V) (Table 2).

### 2.2. Bacillus sp. Bbv57 Shares Significant Similarity with Bacillus subtilis

**Figure 1:**
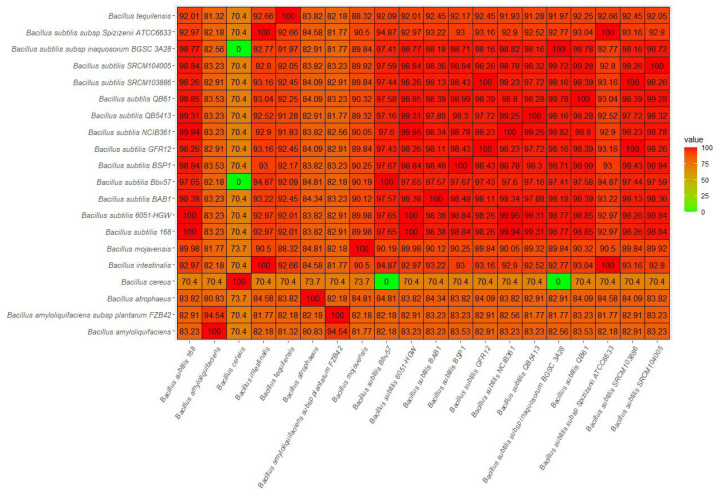
Heatmap of average nucleotide identity (ANI) values for whole genomes of the strain *Bacillus subtilis* Bbv57 and 19 other *Bacillus* species.

**Figure 2:**
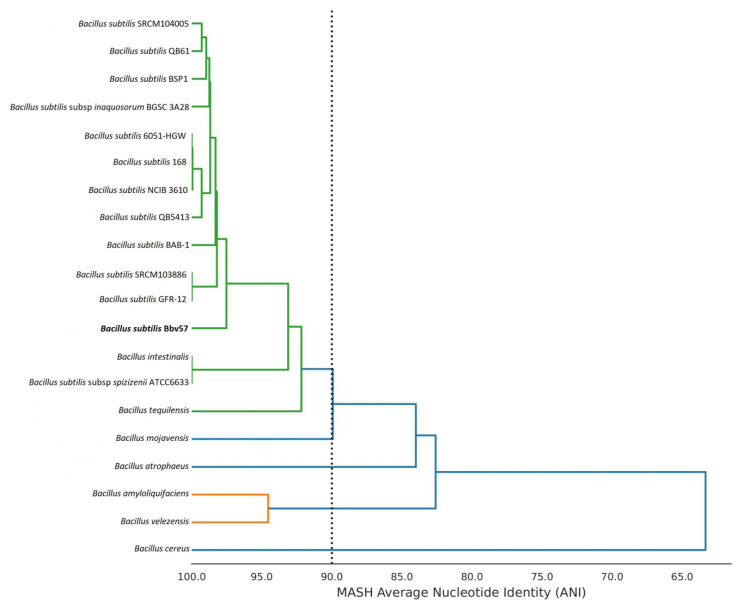
Average nucleotide identity-based phylogenetic tree of 20 different *Bacillus* strains constructed by MASH clustering.

The 16S ribosomal gene similarity was analyzed using a BLAST search against the 16S ribosomal RNA database in CLC workbench 21.0.3; thus, we identified *Bacillus subtilis* IAM 12118 as a top hit with e value 0 and 100% sequence identity. An ANI-based whole genome analysis of 20 different *Bacillus* strains showed that *Bacillus subtilis* Bbv57 was closely related to other 11 different *Bacillus* strains with ANI values of 0.97 or higher (Figure 1). *Bacillus subtilis* Bbv57 and *Bacillus subtilis* BSP1 had the maximum ANI value (97.67%). An analysis of the 16S rRNA sequence and ANI analysis confirmed the identity of Bbv57 as *Bacillus subtilis* (Figure 2).

### 2.3. Bbv57 Harbors Novel Genes Encoding for CAZymes

**Figure 3:**
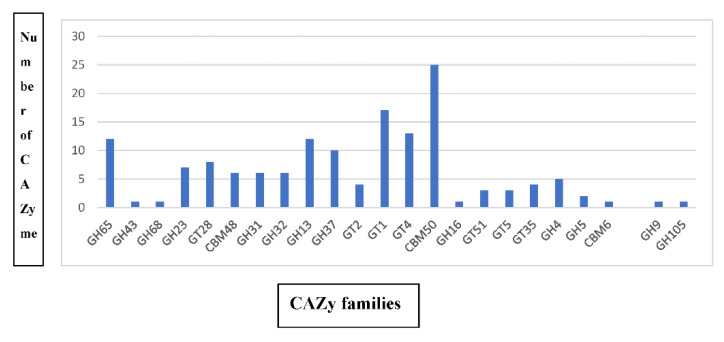
Distribution of the carbohydrate active enzyme (CAZy) family protein identified in the genome of *B. subtilis* Bbv57.

CAZymes are a group of enzymes possessing key roles in carbohydrate metabolism [19], and information on CAZymes is stored in the CAZy database (www.cazy.org, accessed on 16 July 2022). CAZymes are grouped into five different classes, viz., glycoside hydrolases (GHs), glycosyltransferases (GTs), polysaccharide lyases (LPs), carbohydrate esterases (CEs), and auxiliary activities (AAs). A bioinformatic analysis of the Bbv57 genome sequence information identified 65 glycoside hydrolases (GHs), 53 glycosyltransferases (GTs), and 32 carbohydrate-binding modules (CBMs) belonging to the group of CAZymes (Figure 3). *Bacillus subtilis* Bbv57 was found to harbor potential antifungal CAZymes, viz., endo β1,4 glucanase (GH 5), chitinase (GH18), endoglucanase (GH51), and xyloglucanase (GH16), which have the potential to inhibit the growth of plant pathogens. The distribution of CAZymes in the *Bacillus subtilis* Bbv57 suggests that it poses a secondary metabolic potential for this strain.

### 2.4. Bacillus subtilis Bbv57 Harbors Genes Encoding for Antimicrobial Secondary Metabolites

**Figure 4:**
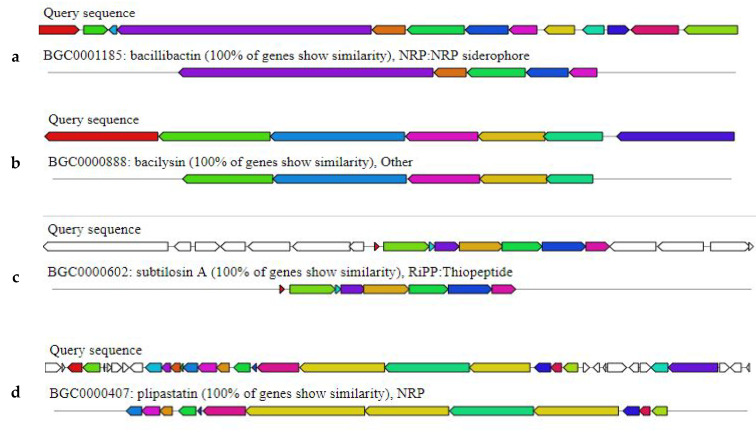

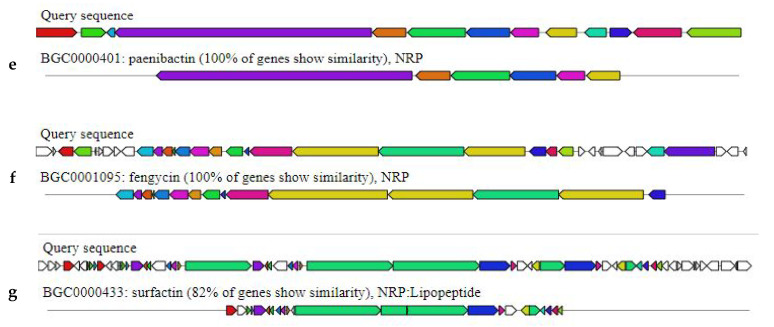
Secondary metabolites’ gene clusters with antimicrobial metabolites in *Bacillus subtilis* Bbv57, identified by antiSMASH 6.0 (**a**) Bacillibactin, (**b**) bacilysin, (**c**) subtilosin A, (**d**) fengycin, (**e**) piplastin, (**f**) paenibactin, and (**g**) surfactin.

The Bbv57 genome was found to harbor genes encoding for novel secondary metabolites having antimicrobial properties. Three gene clusters encoding NRPS (non-ribosomal peptide synthetase), two gene clusters encoding for terpene biosynthesis, one gene cluster for T3PKS (Type III polyketide synthetase), one cluster for CDPS (Cyclodipeptides synthetase), one cluster for sactipeptide biosynthesis, and one gene cluster encoding for bacilysin biosynthesis were all identified in the Bbv57 genome. Among the three gene clusters encoding for NRPS, one cluster was found to exhibit 100% similarity with genes involved in fengycin and piplastin synthesis, another cluster showed 100% similarity with gene clusters involved in bacillibactin and paenibactin synthesis, and the third cluster was found to exhibit 82% similarity with genes involved in the biosynthesis of surfactin. The gene cluster encoding for sactipeptide synthesis showed 100% similarity with subtilosin A (Figure 4).

**Figure 5:**
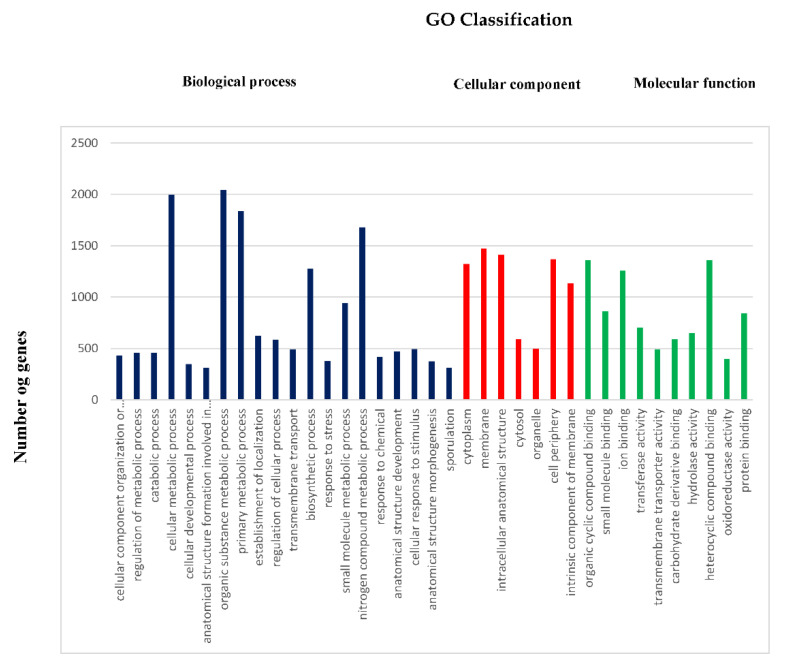
Gene ontology (GO) annotation and functional classification of *Bacillus subtilis* Bbv57. Functional categorization using gene ontology (GO) terms was performed based on the Blastx hits from the nr database using Blast 2 GO annotation in OmicsBox 2.0.10.

A functional categorization by gene ontology (GO) terms was performed based on the Blastx hits from the nr database using Blast 2 GO annotation in OmicsBox 2.0.10. Twenty GO terms belonging to biological processes, 7 GO terms belonging to cellular components, and 10 GO terms belonging to molecular function classes were identified (Figure 5).

### 2.5. Pangenome Analysis of B. subtilis Bbv57

**Figure 6:**
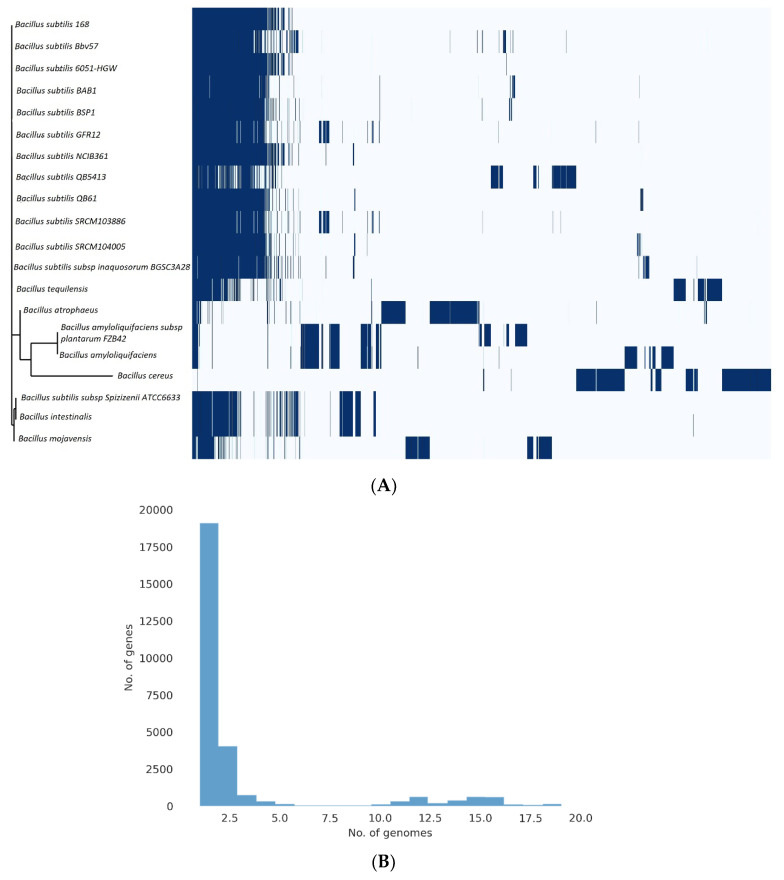
*Bacillus subtilis* pangenome. The pangenomes of six *Bacillus* sp. were determined using the Roary matrix. A total of 28,404 sets of orthologous proteins were found. (**A**) A heatmap showing the gene presence (dark blue) or absence (light blue) in each of the 20 strains. A phylogeny built based on the core genes is shown on the left, and the species names are indicated on the right. (**B**) A histogram displaying the distribution of genomes per gene is found within.

The pangenome model developed by involving 20 strains of *Bacillus*, comprised of 12 strains of *B. subtilis* and 8 strains of *Bacillus* species, indicated a close genetic relationship between Bbv57 and other *Bacillus subtilis* strains. There was only minimal variation in the gene content between the *Bacillus subtilis* strains. The pangenome of 20 *Bacillus* strains consisted of 28404 genes, of which 4281 genes belonged to *Bacillus subtilis* Bbv57. *B. subtilis* Bbv57 was found to harbor some unique gene clusters putatively present in *B. amyloliquefaciens* (Figure 6a,b).

## 3. Discussion

Plant growth-promoting rhizobacteria (PGPR), a group of root-associated bacteria, are involved in modulating plant health and soil fertility through the production of bioactive substances [8]. Among the reported PGPR, *Bacillus* is one of the most exploited bacterial genera for plant growth promotion and biocontrol activity [3]. It suppresses plant pathogens by producing antibiotic metabolites or by stimulating the host’s defense pathways (Van Loon, 2007). Several strains of the genus *Bacillus* have become popular biocontrol agents [20,21]. The author proved that the broad-spectrum activities of *Bacillus* are attributed to its ability to produce a number of secondary metabolites, including antibiotics, volatile HCN, siderophores, chitinase, and ß 1,3-glucanase [22]. It was also demonstrated that *Bacillus* modulates plant growth through the production of IAA, gibberellin, and cytokinin [23]. *Bacillus* harbors various antibiotic biosynthetic genes, viz., iturin A, surfactin, zwittermicin A, and bacillomycin D [24]. Hence, the genome mining of *Bacillus* spp. must be carried out to unravel its genetic potential and to exploit the identified genes/proteins for a disease management program.

In our previous study, we isolated *Bacillus subtilis* Bbv57 from a betelvine’s rhizosphere and found it to exhibit antagonistic activity against a variety of phytopathogens and nematodes [9]. Thin-layer chromatography studies of Bbv57 extracts showed the presence of surfactin and iturin, which were attributed to its inhibitory action against *F. oxysporum* [11]. Similarly, a gas chromatography–mass spectrometry (GCMS) analysis detected the aliphatic hydrocarbons, viz., butanedioic acid, hexadecanoic acid ethyl ester, pentanedioic acid 2-oxo-dimethyl ester, pyrrolo [1,2-a]pyrazine-1,4-dione, hexahydro-3-(2-methylpropyl), pyrrolo [1,2-a]pyrazine-1,4-dione, and hexahydro-3-(phenylmethyl) ester, possessing antifungal, antibacterial, and antinematicidal activity [9]. Thus, the presence of these antimicrobial metabolites in *B. subtilis* Bbv57 might play an important role in its antagonistic activity against phytopathogens. *Bacillus subtilis* can directly prevent the infection of the phytopathogens by releasing the aiiA enzyme, which inactivates acyl homoserine lactone molecules that regulate the expression of virulence genes in plant pathogens [25]. *B. subtilis* naturally colonizes plant roots by forming a thin biofilm that is important for its root colonization and protection. The culture filtrate from the strain Bbv57 significantly reduced the egg-hatching capacity and juvenile mortality of *M. incognita* [9].

In this study, whole genome sequencing combined with bioinformatics analysis shed more light on the molecular basis of the plant growth-promoting and biocontrol abilities of *Bacillus* spp. Bbv57. A detailed sequence analysis of 16s rRNA revealed its 100% identity against *Bacillus subtilis*. This was further confirmed through an alignment of whole genome sequence information against 19 other *Bacillus* strains in the database. An ANI analysis indicated >97% sequence similarity with the *Bacillus subtilis* strain BSP1. An analysis for the presence of CAZymes in the genome of *Bacillus subtilis* Bbv57 identified 65 glycoside hydrolases (GHs), 53 glycosyltransferases (GTs), and 32 carbohydrate-binding modules (CBMs). Specific antimicrobial enzymes, viz., endo β1,4 glucanase (GH 5), chitinase (GH18), endoglucanase (GH51), and xyloglucanase (GH16) were also noticed. The author also reported the antifungal activity of Bbv57 against *Fusarium oxysporum* f. sp. *gerberae* and *Meloidogyne incognita* [9]. In addition, an analysis of the Bbv57 genome for the presence of secondary metabolites showed the presence of antimicrobial genes, which are effective against pathogens. Secondary metabolite gene clusters involved in the biosynthesis of fengycin, piplastin, bacillibactin, paenibactin, surfactin, and subtilosin A were also present in Bbv57. In an earlier study, *Bacillus subtilis* EBPBS4, exhibiting a high level of antagonistic activity against rice sheath blight, was found to harbor 13 antimicrobial peptide genes, viz., iturin A, iturin D, iturin C, surfactin, bacilysin, fengycin, ericin, mycosubtilin, subtilosin, and mersacidin apart from plant growth-promoting genes [5]. The genome of *B. subtilis* PTA-271 possessed secondary metabolites, viz., bacillaene, subtilosin, bacilysin, fengycin, and surfactin, which showed antagonistic activity against a broad spectrum of pathogens [15]. *Bacillus subtilis* genome(s) of various isolates harbor novel genes exhibiting antagonistic activity against plant pathogens and/or the capability of activating induced systemic resistance in plants (Table 3). In our study, the whole genome sequencing of *Bacillus subtilis* Bbv57 identified the genes encoding for novel antimicrobial peptides associated with its biocontrol properties.

## 4. Materials and Methods

### 4.1. Isolation and Maintenance of Bacterial Strain Bbv57

The strain *Bacillus subtilis* Bbv57 with growth-promoting activity and antagonistic activity against phytopathogens and nematodes, isolated from the rhizosphere of a betelvine, was used [9]. The pure culture of the organism was stored in a glycerol stock at −20 °C for further studies. 

### 4.2. Genome Sequencing of Strain B. subtilis Bbv57

A single colony of *Bacillus subtilis* strain Bbv57 was inoculated in Luria–Bertani (LB) nutrient broth and grown overnight at 28 °C in an incubator shaker. The genomic DNA was then extracted from the grown cells using the cetyltrimethyl ammonium bromide (CTAB) method [9]. The grown culture was centrifuged at 6000 rpm for five minutes at 4 °C. The pellet was suspended in 1 ml TE buffer and 0.5 mL butanol and centrifuged at 5000 rpm for five minutes at 4 °C. The pellet was added with 100 µL lysozyme (10 mg/mL) and incubated at room temperature for five minutes. We then added 150 μL of 1% CTAB solution, mixed well, and incubated it at 65 °C for ten minutes. The mixture was extracted with 1 mL of phenol:chloroform mixture, mixed well, and centrifuged at 6000 rpm for 15 min at 4 °C. The aqueous layer was separated, and 0.6 volume of ice-cold isopropanol was added and incubated overnight at −20 °C. The DNA was pelleted by centrifugation at 12,000 rpm for 15 min at 4 °C. The pellet was washed with 70% ethanol, dried under a vacuum for 10 min, and resuspended in 50 µL of TE buffer. The DNA was stored at −20 °C for further use. The integrity of the DNA was confirmed on a 0.8% agarose gel electrophoresis, and its quality and quantity was assessed using a NanoDrop spectrophotometer. The DNA library for genome sequencing was prepared from high-quality genomic DNA using the Nextera XT DNA Library Preparation Kit and TruSeq Nano DNA Kit and sequenced using Illumina platform (PE 2 × 150 bp) (Table 4). The experimental data are available in NCBI (Accession PRJNA794929).

### 4.3. Genome Assembly and Annotation

**Figure 7:**
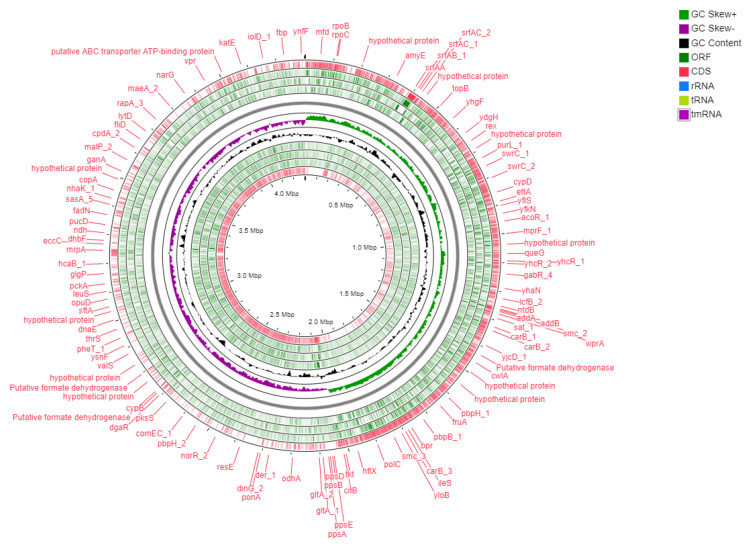
Genome map of *Bacillus subtilis* Bbv57. A circular map of the strain Bbv57 genome was constructed using a CG viewer.

The obtained raw reads were filtered using FastQC version 0.11.9 [35] and sickle version 1.33 [36]. The high-quality adapter-free filtered reads were assembled using SPAdes version 3.9.0 [37] and polished by pilon [38]. The polished sequences were used for reference to guide the scaffolding with *Bacillus subtilis* subsp *subtilis* str 168 (AL009126.3) by ragtag [39]. The gene prediction was performed using Prodigal version 2.6 [40] and annotated using Prokka version 1.12 [41]. A circular map of the strain Bbv57 genome was constructed using a CG viewer [42] (Figure 7). The genes were mapped onto pathways against the Kyoto Encyclopedia of Genes and Genomes (KEGG), the Clusters of Orthologous Groups (COG) classification, and the Gene Ontology (GO) database using OmicsBox 2.0.10 [43].

### 4.4. Molecular Confirmation of Bacillus subtilis Bbv57

The short reads of Bbv57 were processed using CLC Genomics workbench v 21.0.3 (CLC bio, Aarhus, Midtjylland, Denmark). The filtered reads were searched against the 16S ribosomal RNA sequences database using the Blastn program using default parameters. The average nucleotide identity (ANI) between *Bacillus subtilis* Bbv57 and 19 other *Bacillus* strains in the database was calculated using the ANI calculator [44]. An ANI-based phylogenetic tree of 20 different *Bacillus* strains was constructed with MASH clustering [45]. A pangenome analysis of 20 different *Bacillus* strains was carried out to analyze the gene differences using the Roary matrix [46].

### 4.5. Prediction of Genes Encoding for CAZymes and Secondary Metabolites in Bacillus subtilis Bbv57

The predicted protein sequences of *Bacillus subtilis* Bbv57 were aligned with the carbohydrate active enzyme (CAZy) database [19] using OmicsBox 2.0.10 [43]. The secondary metabolite gene clusters were identified using antiSMASH 6.0.1 [47].

## 5. Conclusions

Thus in our study, whole genome sequencing of *Bacillus subtilis* Bbv57 generated 4,302,465 bp and permitted us to assemble the draft genome of *B. subtilis* Bbv57 and to identify its unique features. A detailed bioinformatics analysis of 16S rRNA genes and an ANI analysis revealed its close genetic/sequence similarity to *Bacillus subtilis*. Bbv57 was found to harbor > 100 CAZymes and several antimicrobial secondary metabolites, contributing to its biocontrol activities. A pangenome analysis involving 20 other strains of *Bacillus* revealed that Bbv57 contains 282 unique genes out of its 4281 total number of genes. These 282 unique genes need further exploration. Overall, our study generated molecular evidences for the antagonistic properties of Bbv57 against plant diseases and thus paved way for its large-scale application in sustainable agriculture.

## Figures and Tables

**Table 1:** The general genome feature of *Bacillus subtilis*.

Feature	Value
Genome size (bp)	4,30,2465
G + C content	44.5%
Total number of genes	4363
Total size of protein-coding genes	3,735,486
Protein-coding genes	4281
Average CDs size (bp)	872.57
rRNA number	5
tRNA number	76
tmRNA number	1
Pseudogenes (total)	27

**Table 2:** COG categories of *Bacillus subtilis*.

COG Code	Number	Proportion	Description
J	210	4.91	Translation, ribosomal structure, and biogenesis
A	1	0.02	RNA processing and modification
K	352	8.22	Transcription
L	157	3.67	Replication, recombination, and repair
B	1	0.02	Chromatin structure and dynamics
D	47	1.10	Cell cycle control, cell division, and chromosome partitioning
Y	0	0	Nuclear structure
V	66	1.54	Defense mechanism
T	189	4.41	Signal transduction mechanisms
M	234	5.47	Cell wall/membrane/envelope biogenesis
N	68	1.59	Cell motility
Z	0	0	Cytoskeleton
W	0	0	Extracellular structures
U	56	1.31	Intracellular trafficking, secretion, and vesicular transport
O	106	2.48	Post-translational modification, protein turnover, and chaperons
C	229	5.35	Energy production and conversion
G	335	7.83	Carbohydrate transport and metabolism
E	406	9.48	Amino acid transport and metabolism
F	121	2.83	Nucleotide transport and metabolism
H	136	3.18	Coenzyme transport and metabolism
I	120	2.80	Lipid transport and metabolism
P	285	6.66	Inorganic ion transport and metabolism
Q	92	2.15	Secondary metabolites biosynthesis, transport, and catabolism
R	0	0	General function prediction only
S	1086	25.37	Function unknown
-	117	2.73	Not in COGs

**Table 3:** Functions of genes in the genome of various *Bacillus subtilis* isolates.

Sl. No.	Isolate	Predicted Functions	Reference
1.	*B. subtilis* EBPBS4	Iturin, surfactin, bacillomycin D, fengycin, ericinmycosubtilin, subtilosin, and mersacidin	[5]
2.	*Bacillus subtilis* MBI600	Fengycin, surfactin, bacillaene, bacillibactin, subtilosin A, basilysin, carbohydrate transport and metabolism, aminoacid transport and metabolism, nitrate transporter, magnesium transporter, and potassium uptake	[26]
3.	*Bacillus subtilis* PTA-271	Catecholicsiderophore, surfactin, fengycin, acetoin, 2,3-butanediol, and N-acyl-L-homoserine lactone	[15]
4.	*Bacillus subtilis*	Carbohydrate transport and metabolism, amino acid transport and metabolism, endo-1, 4-ß-glucanase, endo- ß -1,3-,4glucanase, xylose isomerase, and pectatelyase	[27]
5.	*Bacillus subtilis* BAB-1	Non-ribosomal peptide synthetase (NRPS) antibiotics, polyketide synthase (PKS) antibiotics, lantibiotics, surfactin, fengycin, and bacillibactin	[28]
6.	*Bacillus subtilis* XF-1	Antimicrobial lipopeptides (surfactin and fengycin), polyketides (macrolactin and bacillaene), bacillibactin, bacilysin, and chitosanase	[29]
7.	*Bacillus subtilis* CMB32	Antifungal lipopeptides	[30]
8.	*B. subtilis* isolate ME488	Possessing secondary metabolites *ituC*, *ituD*, *bacA*, *bacD*, *mrsA*, and *mrsM*	[31]
9.	*Bacillus subtilis*	Iturin and fengycin	[32]
10.	*Bacillus subtilis* GA1	Lipopeptides	[33]
11.	*Bacillus subtilis* BBK1	Bacillomycin L, plipastatin, and surfactin	[34]

**Table 4:** Genome sequencing information of *Bacillus subtilis*.

Property	Term
Sequencing finishing quality	High quality draft
Libraries used	Illumina paired-end library (2 × 150 bp insert size)
Sequencing platform	IlluminaHiseq
Assemblers	SPAdes
Gene-calling method	Prodigal
BioProject	PRJNA794929
BioSample	SAMN24663524
Source material identifier	*Bacillus subtilis*
Project relevance	Biocontrol

## Data Availability

The data supporting the findings of this study are available within the article. The 16S rRNA gene sequence of the strain was deposited into the GenBank database. The high-quality raw data genome sequence for *Bacillus subtilis* BBv57 was deposited into NCBI under accession SRR17459383.
